# Opti-MSFA: a toolbox for generalized design and optimization of multispectral filter arrays

**DOI:** 10.1364/OE.446767

**Published:** 2022-02-22

**Authors:** Travis W. Sawyer, Michaela Taylor-Williams, Ran Tao, Ruqiao Xia, Calum Williams, Sarah E. Bohndiek

**Affiliations:** 1 Wyant College of Optical Sciences, University of Arizona, 1630 E University Blvd, Tucson, AZ 85721, USA; 2 University of Arizona Health Sciences, University of Arizona,1 670 E. Drachman St. Tucson, Arizona 85721, USA; 3 Department of Physics, Cavendish Laboratory, University of Cambridge, JJ Thomson Avenue, Cambridge, CB3 0HE, UK; 4 Cancer Research UK Cambridge Institute, University of Cambridge, Robinson Way, Cambridge, CB2 0RE, UK; 5 Department of Engineering, Electrical Engineering Division, University of Cambridge, JJ Thomson Avenue, Cambridge, CB3 0FA, UK

## Abstract

Multispectral imaging captures spatial information across a set of discrete spectral channels and is widely utilized across diverse applications such as remote sensing, industrial inspection, and biomedical imaging. Multispectral filter arrays (MSFAs) are filter mosaics integrated atop image sensors that facilitate cost-effective, compact, snapshot multispectral imaging. MSFAs are pre-configured based on application—where filter channels are selected corresponding to targeted absorption spectra—making the design of optimal MSFAs vital for a given application. Despite the availability of many design and optimization approaches for spectral channel selection and spatial arrangement, major limitations remain. There are few robust approaches for joint spectral-spatial optimization, techniques are typically only applicable to limited datasets and most critically, are not available for general use and improvement by the wider community. Here, we reconcile current MSFA design techniques and present Opti-MSFA: a Python-based open-access toolbox for the centralized design and optimization of MSFAs. Opti-MSFA incorporates established spectral-spatial optimization algorithms, such as gradient descent and simulated annealing, multispectral-RGB image reconstruction, and is applicable to user-defined input of spatial-spectral datasets or imagery. We demonstrate the utility of the toolbox by comparing against other published MSFAs using the standard hyperspectral datasets Samson and Jasper Ridge, and further show application on experimentally acquired fluorescence imaging data. In conjunction with end-user input and collaboration, we foresee the continued development of Opti-MSFA for the benefit of the wider research community.

## 1. Introduction

Spectral imaging captures spatially resolved ( x , y ) spectral ( λ ) data from a scene, enabling the composition of different objects to be determined, typically based on their reflectance properties. Spectral imaging has been widely applied across the commercial and scientific sectors, with common applications including materials classification, biomedical imaging and remote sensing [1–4]. Multispectral imaging (MSI) captures spectral data in a limited number of pre-determined wavelength bands, or channels, [5], which is well suited to applications where the spectral features of the object(s) of interest are confined to narrow bandwidths or are slowly varying [6,7]. Better object identification / spectral reconstruction can also be achieved using an optimal subset of wavelength channels [8].

By reducing the number of wavelength channels to be interrogated, MSI enables the realization of compact, high-speed, cost effective spectral imaging systems. In particular, "snapshot" MSI systems are growing in popularity as they can obtain data from targeted wavelength channels, without the need for spatial or spectral scanning [9,10]. A snapshot MSI architecture that is growing in popularity uses the multispectral filter array (MSFAs)—a mosaic of narrowband spectral filters integrated atop a panchromatic image sensor, enabling spectral filtering directly at the pixel level. In combination with fast back-end image processing algorithms, MSFAs enable the reconstruction of the imaged scene within discrete wavelength channels. The most prevalent example of an MSFA is the ubiquitous red, green, blue (RGB) Bayer filter [11]. Commonly referred to as a color filter array (CFA), this 2x2 mosaic includes three broad bandwidth spectral filters (one red, one blue and two green filters), used to mimic the trichromatic human vision system. Further general MSFA examples include: 4-channel RGB with single near-infrared (NIR) channel [12] and 4-channel RGB with single panchromatic channel [13,14]. These implementations all integrate transmissive filter (bandpass) elements, yet subtractive (notch) MSFAs have also been demonstrated, for example the CYYM (one cyan, two yellow, and one magenta filter) MSFA [15–17]. Nonetheless, customization of MSFAs is limited, with wavelength channels either selected empirically or arranged as a series of linearly spaced bandpass filters with similar bandwidths.

Unfortunately, these application-agnostic MSFAs, where wavelength channels are not correlated with the spectral features within the scene are sub-optimal [18], resulting in low signal-to-noise ratio, limited useful spectroscopic information, redundant channels and low optical throughput [19]. Customized MSFAs offer significant improvements in spectral identification, optical throughput and maximizing the amount of available information detected [5,20], yet their complexity (and hence cost) of production—underpinned by micro-patterned thin-film interference (Fabry-Perot) filters [21]—is determined by the number of channels, spectral response characteristics, pixel dimensions and mosaic pattern. Optimizing MSFA design is therefore crucial for maximum information extraction and cost-effectiveness. MSFA optimization is inherently challenging, as there is a strong mutual correlation between different design variables, including: the selection of spectral channels; the spectral filter responses (lineshapes); the number of channels; the spatial distribution of the channels on the sensor; and the spatial and spectral reconstruction methods. As such, a challenging multi-parametric optimization is required to determine the optimal MSFA design.

A number of studies have focused on the optimization of different aspects of MSFAs, optimizing either the spectral sensitivity functions of the wavelength channels or the spatial distribution of channels on a periodic mosaic pattern [9,22,23]. Unfortunately, these methods treat the spatial and spectral aspects separately, thus cannot ensure the optimal MSFA design due to the mutual correlation between the spatial and spectral content. Other methods aim to apply new spectral transmittance functions to the filters to enhance information content, for example, using a radial basis or Fourier basis functions [24,25]. However, these spectral sensitivity functions are challenging and costly to fabricate using existing manufacturing methods, therefore limiting their potential. Several approaches to joint optimization have been implemented in recent years with varying levels of success, these include: deep convolutional networks for CFAs [26,27], sparsity-based methods [28], as well as interior-point optimization [29]. While these continuous optimization methods show promise, the issue of MSFA manufacturing constraints arise. Other approaches instead focus on subselecting filters from available candidates during the joint optimization. For example, near-optimal MSFA design was achieved using a heuristic global search algorithm [30].

In addition to optimizing the spectral response of the channels and the spatial arrangement, the downstream processing of signal reconstruction and object classification must be considered. Spectral reconstruction refers to recovering a higher resolution spectrum from a discrete series of channels. In the simplest case, this is a linear mapping between the channel responses and the original spectrum, for example, using a pseudo-inverse approach [31] or linear regression-based methods [32]. Deep learning has also shown significant promise when applied to accurate spectral reconstruction [33–35]. Finally, using the detected signal, object classification is done by assessing the total signal and decomposing it into a summation of individual component signals (endmembers), known as spectral unmixing.

An important aspect of MSFA design and optimization is the selection of the cost or merit function. The accuracy of spectral unmixing by calculating the mean-squared error (MSE) is commonly used to assess the performance of a particular design for hyperspectral image reconstruction [6,26]. However other ways to measure performance exist as well, for example signal to noise ratio and dynamic range [25], the reconstruction of an RGB image [23], or target classification accuracy [22]. In general, the selection of cost function will drastically impact the resulting MSFA design and should be chosen carefully to ensure concordance with the overall aim and requirements of the design. Ultimately, effective optimization of MSFAs must take into account every step of the detection and processing pipeline.

While an abundance of design and optimization techniques for MSFAs have been developed over the past two decades, there is still no widely accessible and generalized toolbox for use by the wider scientific or commercial community to develop MSFAs and benchmark new approaches. Here, we present Opti-MSFA: an open-access Python-based centralized toolbox for MSFA design, development and optimization, as shown in Code 1 [36]. Features of the toolbox include: integration of different optimization routines for fast evaluation of optimal MSFAs for different datasets; generalizability to user-provided datasets, which facilitates easy comparison of different methods; the ability to freely adjust, prioritize and analyze the effect of different design parameters; and the output of results from the entire pipeline. The code is publicly accessible and enables developers to add / modify the optimization techniques to best suit an individual application and compare against gold standard algorithms. Given the widespread utility of MSFAs for customized spectral imaging applications, and growing researcher numbers in the community, Opti-MSFA aims to provide a single standardized platform for powerful MSFA design, optimization, algorithm benchmarking and future development for a wide variety of applications, ranging from remote sensing to biomedical imaging. We acknowledge that there is a host of literature surrounding MSFA optimization, some generalized and some more specialist [37–40]. We have chosen to focus our efforts on a more generalized joint-optimization framework for MSFA design as opposed to often inaccessible and niche works, in order to be of most value to the research community. Ultimately, we envisage this communal toolbox to offer users the ability to both determine optimal MSFAs for their targeted applications and benchmark new optimization algorithms, such as machine learning-based, against existing approaches.

## 2. Methods

**Fig. 1.**
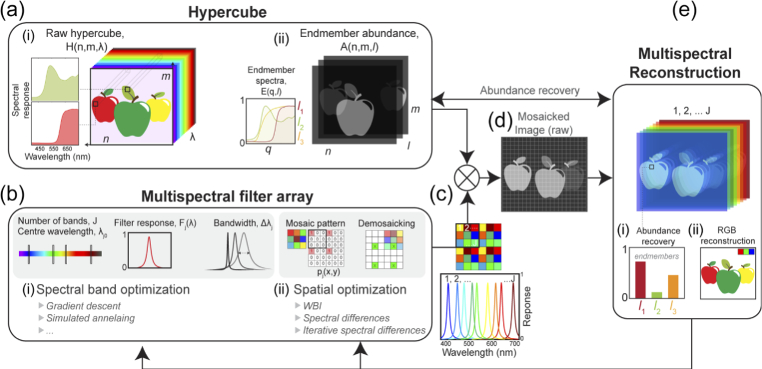
Multispectral Filter Array Optimisation (Opti-MSFA) flowchart and operating overview. (a) Input hypercube datasets; supplied format either (i) raw data or (ii) as an abundance map with endmember abundances. (b) Simulation of MSFA with input parameters through spectral channel (i) and spatial (ii) optimization. (c) The simulated MSFA design for each iteration is multiplied by the input hypercube which generates the raw mosaicked dataset (d). The multispectral image is reconstructed (e) along with endmember abundance (i) and RGB image (ii).

**Fig. 2.**
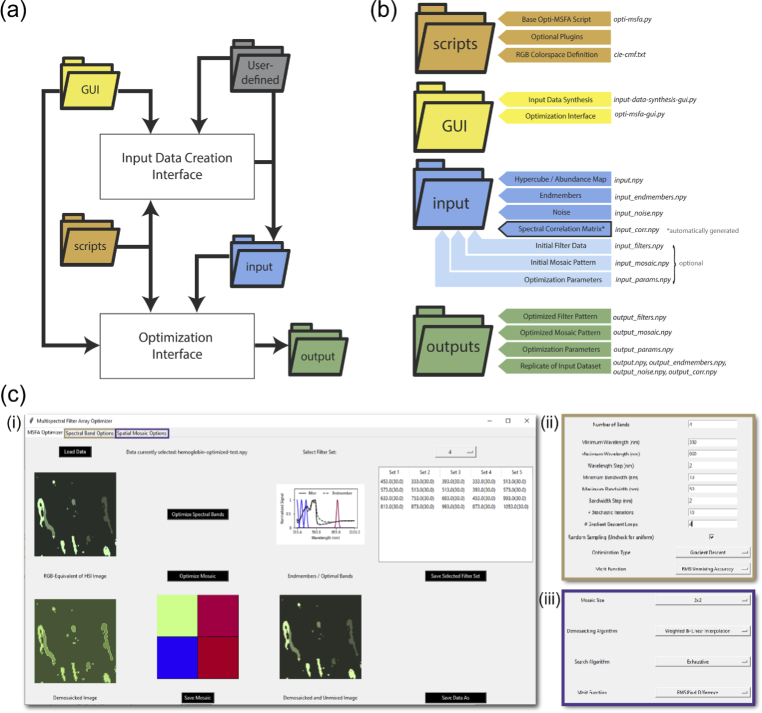
Opti-MSFA toolbox overview. (a) Organization and folder architecture. (b) Hierarchical structure of the Opti-MSFA toolbox. (c) Screenshot of the toolbox GUI (i) highlighting the spectral channel optimization input options (ii) and spatial optimization options (iii).

Opti-MSFA was developed using Python 3 using in-built optimization functions. An overview of the optimization process is illustrated in Fig. 1 and the architecture of the Opti-MSFA code (toolbox structure) is shown in Fig. 2. The program input is a hypercube of spatial (x,y) and spectral ( λ ) data, which can be provided either as raw HSI data, or from an abundance map with separate endmember data, where the endmembers are the spectra that are found within the scene at the prescribed abundances. Irrespective of the hypercube format, endmember data is required for spectral unmixing. Gaussian distributed spatial noise can also be added. Given a set of spectral filter responses, the raw MSFA signal is simulated for each spectral channel, using the hypercube input. The individual channels are arranged according to a given mosaic pattern (spatial arrangement), which is then demosaicked into a full-resolution image. Finally, the endmember abundances can be unmixed from the demosaicked images, enabling pixel-wise comparison to the original input. With this approach, the full process of MSI is simulated, enabling realistic optimization where the merit function reflects the final image quality. The following sections provide detail on how each step of this pipeline operates and the features that Opti-MSFA provides the user.

### 2.1 Input hypercube

The input to the Opti-MSFA pipeline is a hypercube of spatial and spectral data representing the target, and the endmember spectra for identification. The hypercube can be supplied either as (1) raw data H(x,y,λ) , or (2) as an abundance map for the endmember abundances A(x,y) where the value of A at any given ( n , m ) is an abundance vector representing the relative abundance of L different endmembers. In our notation, we discretize x , y , and lambda into N , M , and Q steps respectively. Thus, H is of dimensions ( N , M , Q ) and A is of dimensions ( N , M , L ). Here, the endmember spectra are represented as a matrix E, of size ( Q , L ). With the abundance map A , the signal at any pixel ( n , m ), the signal is represented as:

(1) H(n,m,λ)=E⋅A(n,m)+n,

where n is a vector representing noise that deviates the value of H from the ideal case.

### 2.2 Simulation of MSFA signal

The MSFA matrix $I_{j}(n,m)$ for J-channels is determined using the MSFA mosaic pattern, spectral filter response for each channel and input hypercube. For the $j^{th}$ -channel, the MSFA matrix becomes

(2) $$I_{j}(n,m)=p_{j}(x,y)\int_{\lambda_{min}}^{\lambda_{max}}F_{j}(\lambda )H(n,m,\lambda )d\lambda ,$$

where, $p_{j}(x,y)$ = 0,1; a value of 1 corresponding to the pixel locations where the filters for the $j^{th}$ -channel are placed, 0 otherwise. $F_{j}(\lambda )$ is the normalized filter spectral response defined between the wavelength limits $\lambda_{min}$ and $\lambda_{max}$ , and H is the input hypercube.

### 2.3 Demosaicking

For a set of raw responses for J channels, full resolution images for each channel are recovered using demosaicking. The toolbox features three different demosaicking algorithms: weighted bi-linear interpolation, spectral differences, and iterative spectral differences [41,42]. This is by no means exhaustive: for additional details on alternate demosaicking processes we direct the reader to the literature [43].

*Weighted bi-linear interpolation* (WBI) is one of the most common and simplest forms for demosaicking. To employ this, the binary image, $p_{j}$ , for each image can be related to the demosaicked MSFA image by

(3) $${\tilde{I}}_{j}(n,m)=I_{j}(n,m)⊙p_{j}(n,m),$$

where $I_{j}$ is the full resolution MSFA image and ⊙ represents the Hadamard (element-wise) product. This implies that the channel response for each pixel where the filter is placed is equal to the response of the full-resolution MSFA image at that pixel. The estimate of the full resolution image for the $j^{th}$ -channel, ${\hat{I}}_{j}$ , is then given as

(4) $${\hat{I}}_{j}(n,m)={\tilde{I}}_{j}(n,m)*B,$$

where B is the weighted, normalized bi-linear interpolation kernel. Ultimately, after applying the convolution operation in Eq. (4), the result is the estimated full resolution MSFA image I(n,m,j) .

*Spectral differences* takes spectral correlation between any two channels into account. For this algorithm, the channel difference $\Delta_{kj}$ between a pair of channels ( k , j ) at each pixel is given as

(5) $$\Delta_{kj}(n,m)=\hat{I_{k}}(n,m)⊙p_{j}(n,m)-{\tilde{I}}_{j}(n,m),$$

where $\hat{I_{k}}(n,m)$ is the interpolated result of channel k using WBI, which is then projected onto values for the known channel k . Next, a full resolution channel difference is obtained via WBI,

(6) $${\hat{\Delta }}_{kj}(n,m)=\Delta_{kj}(n,m)*B.$$

Finally, for each channel k , the estimation of ${\hat{I}}_{j}(n,m)$ is given as

(7) $${\hat{I}}_{j}(n,m)=\sum_{k=1}^{K}[({\tilde{I}}_{j}(n,m)-{\hat{\Delta }}_{kj}(n,m))⊙p_{j}(n,m)].$$

*Iterative spectral differences* builds on spectral differences by further taking into account correlation between two channels, which can be strong when the spectral responses are close. To do so, a number of iterations, G , for a channel pair ( k , j ) is calculated as

(8) $$G_{kj}=\exp\left(-3\sigma_{kj}\right).$$

$\sigma_{kj}$ is calculated for a filter pair ( k , j ) by finding the covariance matrix between the signal for each filter when the average spectral value composing H is received. The factor of three is by convention. The algorithm proceeds as above, except for each step of the iteration $t<G_{kj}$ ,

(9) $$\Delta_{t}^{kj}(n,m)={\hat{I}}_{k}^{t-1}(n,m)⊙p_{j}(n,m)-{\tilde{I}}_{j}(n,m),$$

(10) $${\hat{\Delta }}_{t}^{kj}(n,m)=\Delta_{t}^{kj}(n,m)*B,$$

(11) $${\hat{I}}_{j}^{t}(n,m)=\sum_{k=1}^{K}[({\tilde{I}}_{j}(n,m)-{\hat{\Delta }}_{kj}^{t}(n,m))⊙p_{j}(n,m)].$$

Note that $\sigma_{kj}$ is defined by the spectral content of the object, thus this algorithm is dependent on *a priori* knowledge, and hence reconstruction accuracy is conditional upon this. Clearly, the choice of demosaciking algorithm depends on the intended use of the MSFA and how much *a priori* knowledge is available.

### 2.4 Abundance recovery

With a full resolution demosaicked MSFA image, the abundance map of each endmember is estimated using non-negative least squares (NNLS) unmixing for each pixel, expressed below as [44]:

(12) min||E⋅A(n,m)−I(n,m)||;a≥0.

Recalling that E is a matrix of endmember responses, where each column represents a single endmember signature. For the case of an MSFA, the endmember spectrum will be the "pure" spectrum multiplied by the filter response. A(n,m) is the endmember abundance vector for pixel ( n , m ), and I(n,m) is the recorded MSFA spectral signature. This can be solved using a number of different minimization algorithms that are readily available in any numerical programming language. In Opti-MSFA, Python’s built-in least squares minimization routines are used to solve the NNLS problem. The estimated abundance vector at pixel ( n , m ) is given as $\hat{A}(n,m)$ .

### 2.5 Spectral channel optimization

For spectral channel optimization, the merit function used is defined as the "unmixing accuracy", which is the root-mean-square (RMS) difference between the ground-truth abundance map ( $A_{o}(n,m)$ ) and the abundance map recovered when using the set of J filter responses for each pixel ( $A^{\prime }(n,m)$ ),

(13) $$\epsilon_{spec}^{2}=\frac{1}{NM}\sum_{n=1}^{N}\sum_{m=1}^{M}(A_{o}(n,m)-A\prime (n,m;p{\left(x,y)=1)\right)}^{2},$$

where A′(n,m;p(x,y)=1) is calculated prior to demosaicking, thus each pixel is assumed to have every filter response represented equally ( p(x,y)=1 ).

**Fig. 3.**
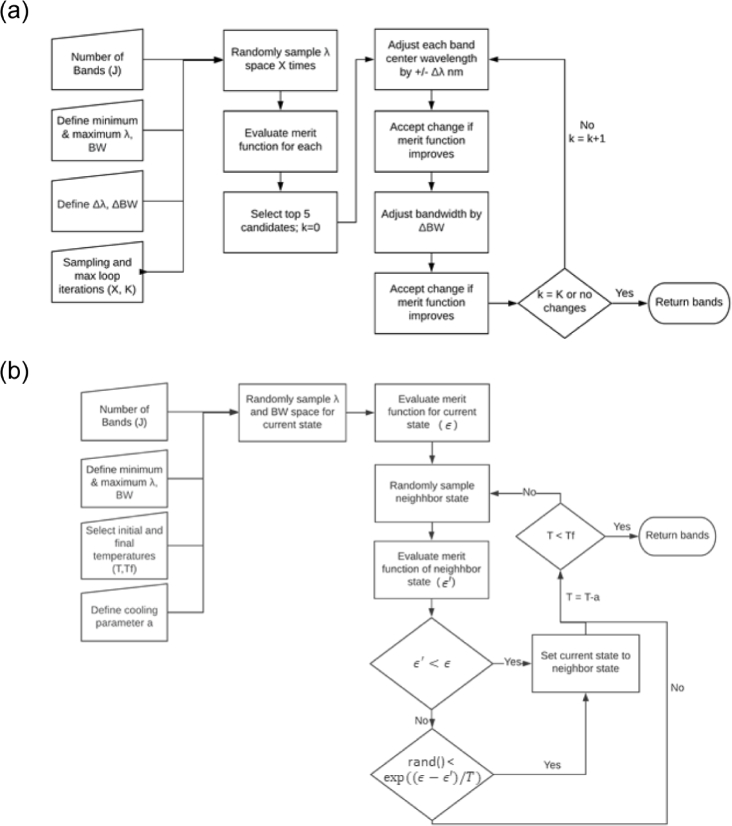
Flowcharts of the optimization algorithms used within the toolbox. (a) Gradient descent optimization and (b) simulated annealing optimization.

Opti-MSFA features two spectral optimization algorithms: gradient descent and simulated annealing (Fig. 3). This optimization step aims to find the spectral sensitivity functions of each filter that will ensure the best recovery of the full resolution spectra.

*Gradient descent* is a popular method used to minimize a function through iterative directional steps [45]. Our implementation begins by initializing all bandwidths at half the prescribed minimum and maximum values. For J spectral channels, the central wavelengths are then initialized by drawing J random samples without replacement from an array defining the wavelength space. This is repeated and the merit functions for each iteration are recorded. The top five are then selected as candidates for gradient descent optimization. For each of the top five candidates, the gradient descent algorithm iteratively adjusts the center wavelengths by ± 2 nm and re-evaluates the merit function. If the merit function is improved, the center wavelength is updated. This then proceeds for the bandwidth and iteratively cycles between center wavelength and bandwidth adjustment until convergence is reached—change in bandwidth or center wavelength is negligible, or the maximum number of iterations has been achieved (set by the user).

*Simulated annealing* models the physical process of annealing and is used to determine the final resting point of a system. In contrast to gradient descent, it is typically more effective at determining an approximate global minimum. In traditional annealing, the metal (system) is heated to a high temperature, and as it cools, it settles into the lowest possible energy state [46]. In this analogy, the energy state is an output of the merit function for a given set of channels. The initial temperature of the system, temperature steps and final temperature can be set by the user. For the initial state of the system, the central wavelengths are then initialized by drawing J random samples without replacement from an array defining the wavelength space, and the bandwidths of the system are set to the center of the minimum and maximum bandwidths allowed. The algorithm loops until the final temperature (stop condition) is reached. Next, a neighbor state is randomly defined, with center wavelengths and bandwidths < 10 nm of the current system. A Metropolis function is used to determine if the neighboring state is accepted. If the difference is better, i.e. the merit function has decreased, the new state is set to the neighboring state, and the systems’ temperature does not change. If the difference is worse, the new solution may randomly be accepted with a probability of

$$\exp(-\frac{\epsilon \prime -\epsilon }{T}),$$

where ϵ is the merit function of the current state, ϵ′ is the merit function of the neighbouring state, and T is the temperature of the current state. If this occurs, the temperature of the system is decreased. This will continue until the system reaches the final defined temperature, and the current center wavelengths and bandwidths are selected.

### 2.6 Spatial mosaic optimization

The spatial optimization of Opti-MSFA aims to find the optimal mosaic pattern to ensure the highest demosaicking performance is achieved using an exhaustive search of different mosaic patterns. For each candidate pattern, the raw MSFA signal is demosaicked using the selected method (Section 2.3). The demosaicked result is compared to the ground-truth full resolution MSFA image that would be obtained if each filter were responsive for every pixel; $p_{j}(x,y)=1\forall x,y,j$ . The merit function is evaluated as the pixel-wise RMS difference over J-channels between the demosaicked result using a candidate filter pattern and the ground truth:

(14) $$\epsilon_{spatial}^{2}=\frac{1}{NMJ}\sum_{n=1}^{N}\sum_{m=1}^{M}\sum_{j=1}^{J}{\left(I_{j}(n,m)-{\hat{I}}_{j}(n,m)\right)}^{2},$$

After exhaustively choosing every mosaic combination, the arrangement that yields the best value of the merit function is selected as optimal. Ultimately, the optimal demosaicked result can then be unmixed at each pixel (Eqn. (12)) to yield the global RMS difference between the input abundance map ( $A_{o}(n,m)$ ) and the final result from the optimization pipeline, as defined by,

(15) $$\epsilon_{spec}^{2}=\frac{1}{NM}\sum_{n=1}^{N}\sum_{m=1}^{M}(A_{o}(n,m)-\hat{A}(n,m;p{\left(x,y)=1)\right)}^{2}.$$

In this merit function, $\hat{A}(n,m)$ is calculated by unmixing the demosaicked MSFA result, in contrast to the spectral (Eqn. 13) and spatial (Eqn. 14) merit functions. With these three merit functions available, the optimization can be carried out separately for spectral and spatial content, or jointly. All results in subsequent sections specifying unmixing error refer to the total error (Eqn. 15).

### 2.7 Conversion to RGB

The information acquired through narrowband MSFAs differs significantly from that of the broad overlapping RGB filter responses utilised within conventional white light image sensors. RGB filter response functions mimic that of the human vision system, thus making visualisation of the acquired data appear realistic. For MSFAs this ability is lost and for qualitative visualization purposes narrowband signal-to-RGB conversion is imperative. Opti-MSFA features the ability to convert the MSFA signal to RGB representation. This is based on the CIE definition of the RGB spectral sensitivity curves [47], and is represented mathematically as a conversion from wavelength space to X , Y , Z color space:

(16) $$X=\int F(\lambda )\bar{x}(\lambda )d\lambda ,$$

(17) $$Y=\int F(\lambda )\bar{y}(\lambda )d\lambda ,$$

(18) $$Z=\int F(\lambda )\bar{z}(\lambda )d\lambda .$$

F(λ) is the filter’s spectral signature for a given pixel, and $\bar{x}$ , $\bar{y}$ , $\bar{z}$ are the CIE color matching functions representing the spectral response of the cone cells in the human eye [47]. X , Y and Z can be normalized to so-called chromaticity coordinates as

(19) $$x=\frac{X}{X+Y+Z};y=\frac{Y}{X+Y+Z};z=1-x-y.$$

Finally, a color space is selected, which is defined by three colors making up the RGB gamut, including white point. This can then be used to convert [x,y,z] to [RGB] by inverting the following matrix relationship:

(20) $$\begin{array}{c} \left[\begin{array}{c} x\\ y\\ z \end{array}\right]=\left[\begin{array}{ccc} x_{r} & x_{g} & x_{b}\\ y_{r} & y_{g} & y_{b}\\ z_{r} & z_{g} & z_{b} \end{array}\right]\left[\begin{array}{c} R\\ G\\ B \end{array}\right]. \end{array}$$

The choice of color space generally depends on how the color is being displayed. Opti-MSFA uses the sRGB color space, and we select the CIE Illuminant D65 as the white point [48]. The xyz values for Eqn. 20 are shown in Table 1.

**Table 1.** Chromaticity Coordinates Used for Conversion to RGB.

	x	y	z
R	0.640	0.330	0.030
G	0.300	0.600	0.100
B	0.150	0.060	0.790
White (Illuminant D65)	0.313	0.329	0.358

To evaluate the visual performance of the MSFA - the representation in RGB for visualisation purposes - the demosaicked MSFA signal for each channel is represented spectrally as the filter response for each channel then converted to RGB space to yield the RGB representation of the MSFA signal as ${\hat{I}}_{RGB}$ . The MSFA RGB representation is then compared to the RGB-equivalent input hypercube, which is obtained using the procedure in Eqns. 16 – 20 on the input hypercube signal for each pixel, $H_{RGB}$ . The spatial merit function can thus be written as:

(21) $$\epsilon_{spatial,RGB}^{2}=\frac{1}{3NM}\sum_{n=1}^{N}\sum_{m=1}^{M}\sum_{j=3}^{J}{\left(H_{j,RGB}(n,m)-{\hat{I}}_{j},RGB(n,m)\right)}^{2}.$$

Note that the J channels in the above representation simply correspond to the red, green and blue channels. This additional feature can enable comparison of the visual performance of MSFAs for both quantitative and visual performance.

### 2.8 Reference data

Two publicly available reference datasets were used. The Samson dataset is composed of 952 x 952 pixels, with 156 spectral channels ranging from 401nm to 889 nm with a spectral resolution of 3.13 nm. To reduce the computational cost, a sub image of 95 x 95 pixels is extracted from the image starting at the (252,332) pixel index. Three endmembers are present (soil, tree, water). The Jasper ridge dataset is composed of 512 x 614 pixels with 224 spectral channels between 380 nm to 2500 nm at a spectral resolution of 9.46 nm. A sub image of 100 x 100 pixels is extracted from the (105,269) pixel index to reduce the computational cost. Channels 1-3, 108-112,154-166, and 220-224 were removed due to the atmospheric effects as is commonly done. This results in 198 channels. Four endmembers are in this dataset (road, soil, water and trees). The ground truth abundance map was spatially truncated to the appropriate size and region in both cases, and the endmember content was also adjusted to remove the relevant channels for the Jasper ridge dataset.

A further experimental fluorescence dataset was used as a test case, given the interest in the application of MSI in biomedical fluorescence imaging. The data was acquired with a benchtop spectral imaging system, using a multi-LED source for illumination, as previously published [49]. Briefly, a near infrared hyperspectral linescan sensor with 72 spectral channels between 600–900 nm (IMEC, Belgium) was used for detection. Mixtures of four fluorescent dyes (AlexaFluor (AF) 610, 647, 700 and 750) with a range of quantum yields and variable overlap between emission spectra were imaged in well plates and compared to data from pure solutions acquired at 10 μM . We test three combinations: AF610 with AF657, AF610 with both AF647 and AF700, and finally a mixture of all four dyes. In every case, the dyes are mixed with equal concentrations of 10 μM .

## 3. Results and discussion

### 3.1 Demonstration using standardized HSI datasets

**Fig. 4.**
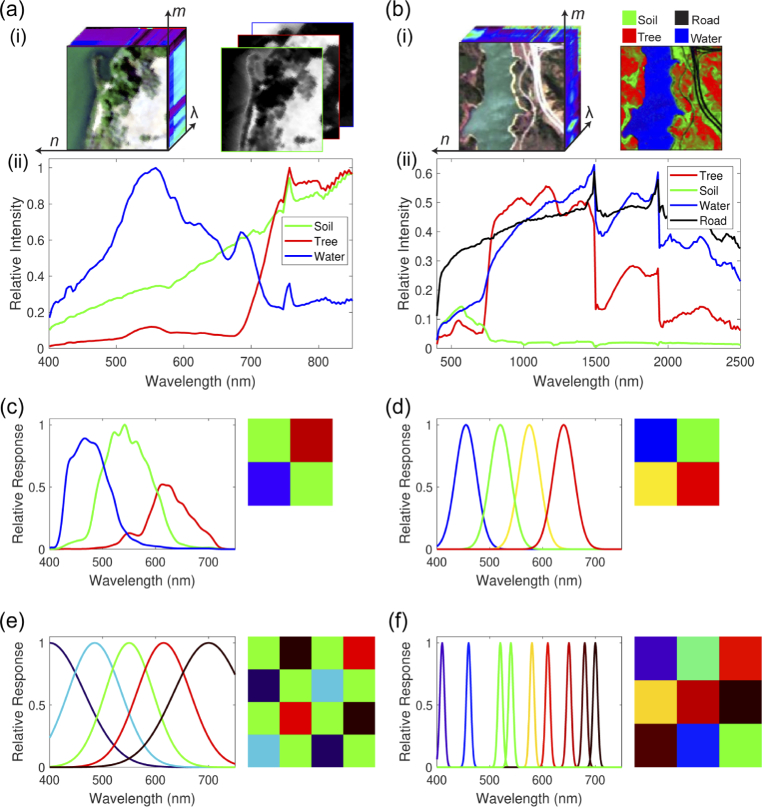
Input hyperspectral datasets for Opti-MSFA. (a) Samson reference dataset; (i) 3D hypercube and abundance map with three different endmembers (ii) soil, trees and water. (b) Jasper Ridge reference dataset; (i) 3D hypercube and abundance map with four different endmembers (ii) soil, trees, water and roads. Reference MSFA designs used to compare the results obtained from Opti-MSFA, for: (c) 3 channels (2x2, RGB) [51], (d) 4 channels (2x2, RGYB) [52], (e) 6 channels [24], and (f) 9 channels (3x3) [18].

To evaluate Opti-MSFA performance, we designed optimal MSFAs for unmixing endmembers in two real-world reference datasets and compared the results against published MSFA designs for the same datasets. The two standardized datasets are the Samson and Jasper Ridge HSI datasets [50] and Opti-MSFA results were compared to 4 MSFA designs (Fig. 4, Table 2):

1. A commercial 2x2 RGGB CFA featuring off-the-shelf RGB filters [51].
2. An empirically-designed 2x2 MSFA, similar to a Bayer filter (RGGB) but with a yellow filter instead of an additional green (RGYB) [52].
3. A five-channel MSFA with center wavelengths and bandwidths optimized for spectral recovery; the spatial distribution of the filters on the mosaic was produced using the binary tree pattern approach [24].
4. An application-independent 3x3 MSFA design that was optimized on three reference HSI datasets that are not used in this evaluation (CAVE, Harvard, ICVL) [18]; all bandwidths are fixed at 10 nm.

**Table 2.** Characteristics of the MSFA / CFA designs that are used to benchmark the toolbox. Bandwidths are defined as full-width at half ${\mathrm{maximum}}^{1}$ ; defined as Gaussian standard ${\mathrm{deviation}}^{2}$ .

Number of Channels	Mosaic Pattern	Center Wavelength (nm)	Bandwidths (nm)
3	2x2	Off-the-shelf filter for commercial cameras [51]	
4	2x2	[455, 575, 520, 640]	[47, 47, 47, 47]^1^
5	Binary Tree	[400, 485, 550, 615, 700]	[153, 117, 106, 117, 153]^1^
9	3x3	[410, 580, 680, 520, 650, 460, 610, 700, 540]	[10, 10, 10, 10, 10, 10, 10, 10, 10]^2^

These four MSFA designs were input into the processing pipeline and used to estimate abundance maps and associated unmixing error for the Samson and Jasper Ridge datasets. The Opti-MSFA pipeline was then used to produce an optimal MSFA design for each dataset. The optimization was first conducted for the visible wavelength range (400–700 nm), for ease of comparison against the reference MSFA designs, and then repeated for the entire wavelength span of the datasets for completeness. The RMS accuracy of the demosaicked and unmixed result was compared to the ground-truth abundance map. The optimization pipeline was repeated for 3, 4, 6, and 9 MSFA channels for both Samson and Jasper Ridge datasets, for comparison to the prior MSFA designs (Table 2). Note that 6-channel MSFA is used to compare against the 5-channel binary tree MSFA since all mosaics were optimized using a unit-cell approach in Opti-MSFA. The spectral optimization was conducted using the gradient descent algorithm with weighted bi-linear interpolation for demosaicking. Other parameters used for the optimization are listed in Table 3.

**Table 3.** Overview of the Input Parameters Used in the Optimization of 3-,4-,6-and 9-channel MSFAs for the Samson and Jasper Ridge Datasets.

Dataset	Wavelength Range (nm)	Bandwidth (nm) [minimum / maximum]			
		3 Channel	4 Channel	6 Channel	9 Channel
Samson (visible)	400 - 700	[10 / 50]	[10 / 50]	[10 / 20]	[10 / 20]
Samson (full range)	400 - 889	[10 / 50]	[10 / 50]	[10 / 50]	[10 / 50]
Jasper Ridge (visible)	400 - 700	[20 / 50]	[20 / 50]	[20 / 50]	[20 / 50]
Jasper Ridge (full range)	380 - 2500	[20 / 50]	[20 / 50]	[20 / 50]	[20 / 50]

**Fig. 5.**
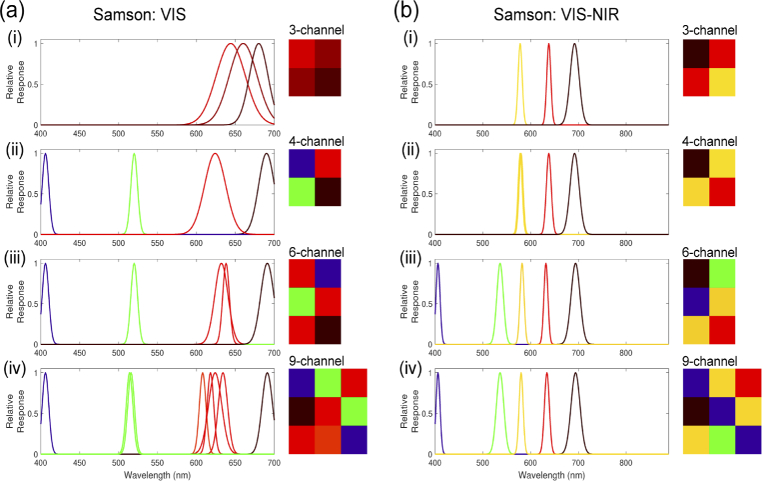
Optimal MSFA designs for the Samson dataset using, (a) only the visible (VIS) wavelengths and (b) the full range (VIS–NIR). Each sub-panel (i–iv) shows the relative spectral response and spatial arrangement of the channels for the 3-,4-,6-,9-channel MSFA respectively.

**Fig. 6.**
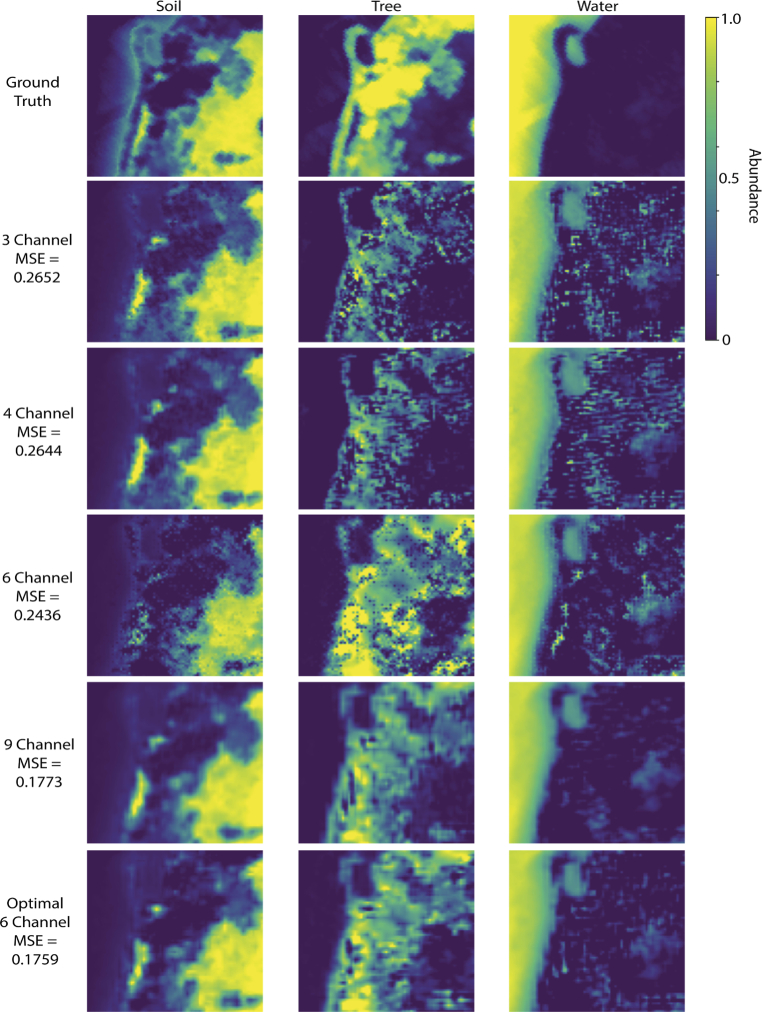
Resulting abundance maps of the reference MSFAs listed in Table 2, as well as the optimal 6-band MSFA Shown in Table 4 compared to ground truth for Samson dataset (full range).

**Fig. 7.**
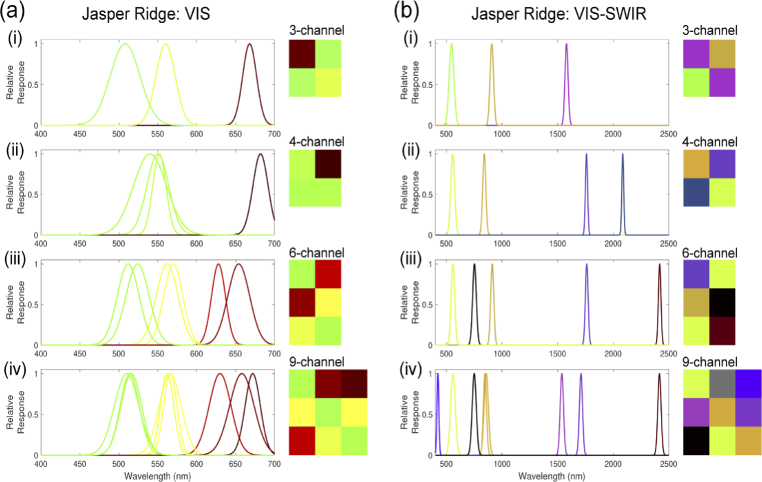
Optimal MSFA designs for the Jasper Ridge dataset using, (a) only the visible (VIS) wavelengths and (b) the full range (VIS–SWIR). Each sub-panel (i–iv) shows the relative spectral response and spatial arrangement of the channels for the 3-,4-,6-,9-channel MSFA respectively.

**Fig. 8.**
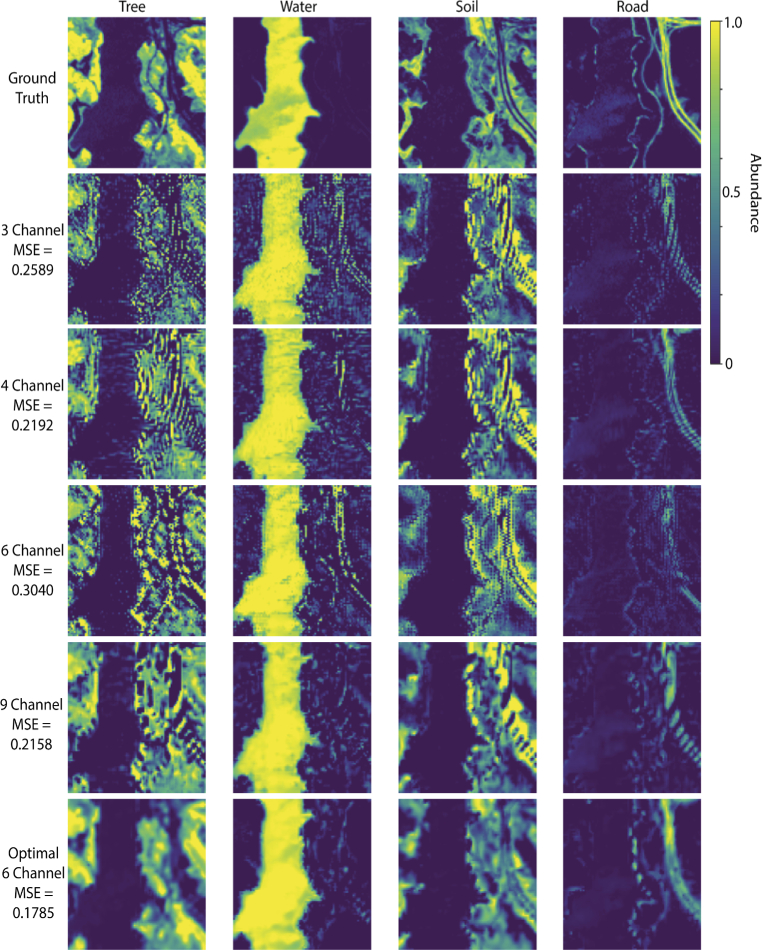
Resulting abundance maps of the reference MSFAs listed in Table 2, as well as the optimal 6-band MSFA Shown in Table 4 compared to ground truth for Jasper Ridge dataset (visible spectrum).

The Opti-MSFA output for the Samson dataset (Fig. 5, Table 4) shows excellent performance (low unmixing error) when compared against other published MSFA designs for recovering the full resolution HSI image from the MSFA data with 4 and 6 channels. We see that the 6-channel design performs the best in the visible range. Also, as expected, the performance is further improved when including the NIR region of the spectrum. With the addition of this extended portion of the waveband, even the 6-channel design performs higher than the published 9-channel visible MSFA. Fig. 6 shows the resulting abundance map for the optimal MSFA compared to the published reference MSFAs for the Samson dataset. Similarly, the Opti-MSFA output for the Jasper Ridge dataset (Fig. 7, Table 5) performs well when compared against other published MSFA designs for recovering the full resolution HSI image from the MSFA data. In this dataset, a large increase in performance is observed when including the infrared spectrum, which implies that a large number of spectral features can be found in that wavelength region. Fig. 8 shows the resulting abundance maps for the optimal MSFA compared to the published reference MSFAs for the Jasper Ridge dataset.

**Table 4.** Characteristics and Unmixing Errors of the Optimal 3-,4-,6-and 9-Channel MSFAs for the Samson Dataset Generated by the Opti-MSFA Toolbox, with Comparative Reference Dataset (Table 2)

	No. of Channels	Center Wavelengths (nm)	Bandwidths (nm)	Unmixing Error
Opti-MSFA (visible)	3	[644, 660, 680]	[44, 42, 48]	0.3177
	4	[690, 520, 406, 624]	[24, 10, 10, 32]	0.2336
	6	[406, 632, 638, 638, 520, 691]	[10, 20, 10, 10, 10, 20]	0.2278
	9	[608, 624, 618, 406, 406, 634, 691, 514, 516]	[10, 20, 10, 10, 10, 14, 16, 10, 10]	0.2463
Opti-MSFA (full range)	3	[578, 638, 692]	[10, 10, 22]	0.1868
	4	[638, 578, 580, 692]	[12, 10, 10, 22]	0.1882
	6	[536, 694, 632, 582, 406, 582]	[16, 22, 10, 10, 10, 10]	0.1769
	9	[580, 406, 580, 580, 536, 694, 634, 406, 580]	[10, 10, 10, 10, 18, 22, 12, 10, 10]	0.1759
Reference (Table 2)	Unmixing Error: 3-channel = 0.2652; 4-channel = 0.2644; 6-channel = 0.2436; 9-channel = 0.1773			

**Table 5.** Characteristics and Unmixing Errors of the Optimal 3-,4-,6-and 9-Channel MSFAs for the Jasper Ridge Dataset Generated by the Opti-MSFA Toolbox, with Comparative Reference Dataset (Table 2)

	No. of Channels	Center Wavelengths (nm)	Bandwidths (nm)	Unmixing Error
Opti-MSFA (visible)	3	[668, 508, 560]	[21, 45, 29]	0.1895
	4	[552, 550, 682, 540]	[20, 33, 23, 50]	0.2326
	6	[628, 562, 570, 512, 654, 524]	[21, 33, 29, 33, 33, 35]	0.1785
	9	[514, 630, 566, 562, 510, 672, 566, 658, 516]	[29, 33, 33, 20, 37, 23, 20, 37, 31]	0.1638
Opti-MSFA (full range)	3	[546, 1580, 908]	[50, 33, 37]	0.0919
	4	[1760, 840, 556, 2086]	[23, 33, 41, 20]	0.0990
	6	[558, 752, 1762, 2418, 912, 558]	[39, 39, 29, 27, 33, 37]	0.1027
	9	[1538, 858, 846, 558, 2416, 558, 1710, 423, 750]	[35, 45, 33, 50, 35, 50, 33, 20, 43]	0.1121
Reference (Table 2)	Unmixing Error: 3-channel = 0.2589; 4-channel = 0.2192; 6-channel = 0.3040; 9-channel = 0.2158			

In the optimization results with a higher number of spectral bands for both data sets, some of the resulting spectral bands are nearly overlapping (Fig. 5, 7). This redundancy occurs when a high level of accuracy is obtained with the selected spectra, and the replication of bands produces a higher level of spatial resolution during demosaicking. In previous studies, we have shown that increasing the number of unique spectral bands will have a limit to the increase in spectral unmixing, and, in some cases, can actually reduce the accuracy [6]. Thus, this redundancy is consistent with these previous observations, and indicates that the optimization technique accounts for this by distributing the available channels to increase the demosaicking performance. It is interesting to note that the reference MSFAs include those that are focused primarily on spatial optimization (e.g, equally spaced spectra in the 4-channel and binary tree) and also those focused on spectral optimization (9-channel design). The improvement observed using the joint optimization approach suggests that the best performance is achieved by considering both spatial and spectral variables.

### 3.2 Application to experimentally acquired fluorescence data

**Fig. 9.**
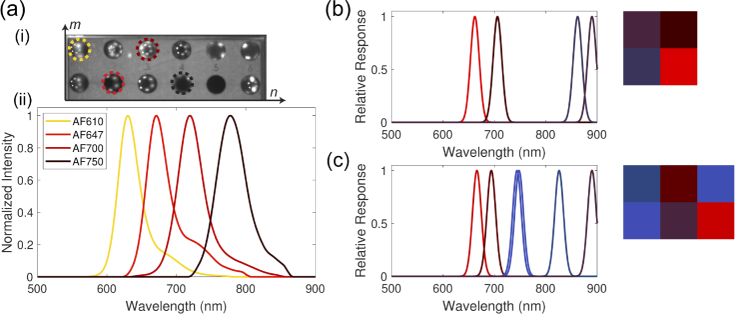
(a) Acquired experimental imaging dataset of a well plate containing four different fluorophore endmembers AF610, AF647, AF700 and AF750 (i) with associated emission spectral signals (ii). Optimal 4 channel (b) and 6 channel (c) MSFAs for detecting the endmembers of the experimentally derived Alexa Fluor dataset.

To further demonstrate the utility of Opti-MSFA, we designed optimal MSFAs to unmix fluorescence spectra from mixtures of fluorescence dyes, based on an experimentally measured spectral imaging dataset. These data provide an important application scenario relevant to biomedical imaging—in particular fluorescent guided surgery—in which to test the accuracy and performance of Opti-MSFA. Input endmembers (Fig. 9(a)) included the fluorophore emission spectra and the light source reflectance spectrum, measured by imaging a white spectralon target [49]. With the raw HSI data from the benchtop experiment used as input for Opti-MSFA, the optimization was run using a minimum bandwidth of 10 nm, maximum bandwidth of 50 nm, for a total of either 4 or 6 channels. We omit the case of 9 channels as we observe that the additional channels results in duplicated spectral channels. As before, spectral optimization was conducted using the gradient descent algorithm, and weighted bi-linear interpolation was used.

Applying the Opti-MSFA optimization to design an MSFA for the experimentally acquired data shows that the results for the 4 and 6 channel designs show remarkable similarly, with essentially four shared filters (Fig. 9). The 6-channel design then has additional filters centered at 744 nm and 748 nm, which essentially overlap. The overall unmixing error of the 4 channel design is 0.0342, which is reduced to 0.0008 for the 6-channel design. This performance improvement is expected—with 5 endmembers, it would be expected to need at least 5 independent measurements to unmix the result. This suggests that the best spectral unmixing performance could be achieved with just one of these two choices. Testing with 9 channels results in additional channel duplication and no further performance increase.

## 4. Discussion

MSFAs enable cost-effective, compact and snapshot MSI for a wide variety of applications. MSFAs are preconfigured with a specific set of spectral channels, hence the design of optimized MSFAs that accurately reconstruct the spectral information contained within a scene using the fewest possible channels is imperative. Prior work has targeted either spectral filter selection or spatial arrangement, with only a small number of joint spectral and spatial optimization approaches reported; the majority also use limited datasets and crucially, are not available for use by the wider community.

In this manuscript, we present the first version of the open-source MSFA design and optimization toolbox Opti-MSFA. Our demonstration on Samson and Jackson Ridge datasets show that some application-independent MSFAs can perform well on specific datasets but not on others. For example, the 9 channel MSFA tested performs the best out of the four reference designs on the Samson dataset but not on the Jasper-Ridge dataset. In contrast, the 6 channel MSFA performs well on the Jasper-Ridge dataset, but not on the Samson dataset. Using our Opti-MSFA tailored designs increases the performance in both cases, showing that ultimately, the best accuracy can be achieved using a custom MSFA. Our application of Opti-MSFA to a raw experimental dataset shows that Opti-MSFA is robust and effective for different data classes by using fluorescence rather than reflectance data, and shows that both can be unmixed from one another using this process. The outputs from these tests demonstrate that Opti-MSFA enables rapid comparison between different MSFA designs, as well as built-in optimization that can produce effective solutions.

The generalizable framework of Opti-MSFA was developed with future integration of additional optimization and reconstruction methods in mind. The first version is capable of optimizing MSFAs for arbitrary input hyperspectral datasets with a host of user-controlled features, however, there remains a number of strategic aims for future development. One clear area is introducing more advanced and varied methods for spectral optimization, merit functions, different unmixing methods, and demosaicking approaches [53–56]. Along these lines, implementing other metrics for performance, including target classification accuracy, signal-to-noise ratio, and dynamic range, would increase the overall utility of the package. Opti-MSFA could also incorporate post-processing techniques for denoising, including median and Gaussian filtering, or machine learning-based methods to incorporate an additional level of image processing that is commonly used with spectral data analysis. Given the abundance of available techniques for these operations, it would be valuable to build up the capabilities of the Opti-MSFA.

Further, because the success and utility of such a toolbox is dependent on community adoption, we foresee development driven in conjunction with the community. For example: incorporating different design and optimization algorithms developed by researchers; benchmarking against methods integrated into Opti-MSFA and posting of results for other end-users; and uploading additional established hyperspectral datasets for others to investigate. Moreover, Python-based Opti-MSFA can be adapted and modified in a relatively straightforward manner to incorporate other powerful Python toolboxes, such as Keras or Tensorflow [57,58], for further improvements in MSFA optimization.

The expansion of the toolbox to incorporate optics and alternate filter functions, represents an important direction for its next iteration. Firstly, in this work, the optical filter functions underpinning the MSFAs are represented by a Gaussian—simply described by the parameters of bandwidth and center wavelength—and hence straightforward to incorporate within an optimization framework. However, the normalized filter response, F(λ) , can be modified by the user. New filter responses would either need to be described by a parameterised function (in order to optimise certain aspects, i.e. center wavelength based on some variable) or through a library of possible functions associated with the filtering mechanism. The former approach can be used to incorporate numerical functions (such as the Lorentzian-described Fabry-Perot resonator), whereas the latter approach can be used for novel filter arrays based on plasmonic, metasurface or diffractive effects [59–62], which derive their filter functions through exhaustive searching within full-wave electromagnetic solvers for a set of specific geometries / materials. The second area of expansion is the incorporation of optical modelling into the toolbox—which would provide an accurate representation of a real-world imaging system. This would include modelling both filter-based optics and camera-based optics. For example, filter response angular sensitivity (i.e. blue shift with increasing angle of incidence), finite aperture of the imaging system, effect of pixel scaling on filter functions [63,64], sensor resolution, silicon responsivity curve of the sensor [37], etc. For example, the authors can envisage a user specifying hardware specific information (i.e. imaging objective f-number, focal length, number of pixels, pixel size etc.) as input variables, which would be incorporated into the optimization through the calculated resultant effect on aspects such as filter response centre wavelength (and bandwidth) blue shift due to ray angle and restriction of number of bands due to pixel limiting spatial resolution. Real-world considerations will typically lead to a reduction in idealised performance characteristics (the current iteration) due to the aforementioned effects. However, these additions increase the real-world robustness of Opti-MSFA and in particular will guide selection of imaging objectives, pixel sizes and filter types through this effective ’ design tolerancing’.

Lastly, manufacturing considerations, such as fabrication tolerances could also be included as boundary limits, while a penalty could be introduced to merit functions when challenging or costly filter designs are considered. Longer term, we could also foresee incorporating polarization-sensitive filter arrays, which are now becoming more prevalent due to the introduction of polarimetric pixel sensors [65]. Additional frameworks, or capability, which include polarization interactions would thus be a valuable addition.

## 5. Conclusion

To address the challenge of spatio-spectral optimization of MSFAs for snapshot MSI, we introduce here Opti-MSFA: an open-access Python toolbox for the community [36]. Opti-MSFA incorporates joint spectral-spatial optimization algorithms, multispectral-RGB image reconstruction, benchmarking, and is generally applicable to user-defined input hyperspectral datasets/imagery. We demonstrate the utility of Opti-MSFA by designing optimal MSFAs for several reference hyperspectral datasets and find that the results compare favourably to other published designs in the literature. We further test Opti-MSFA by designing optimized MSFAs for experimentally acquired fluorescence spectral imaging data, illustrating generalizability. Ultimately, our toolbox has been produced with the research community in mind, and in conjunction with end-user input and collaboration in further developments, Opti-MSFA could find widespread application for MSFA design for scientific and commercial contexts, as well as for the benchmarking of new algorithms.

## Data Availability

Data underlying the results presented in this paper are available in Code 1, Ref. [36]. A tutorial is included in this repository detailing how users can contribute to the code by integrating optimization algorithms, different filtering mechanisms, merit functions, etc.
